# Learning Gaussian graphical models from correlated data

**DOI:** 10.3389/fsysb.2025.1589079

**Published:** 2025-07-03

**Authors:** Zeyuan Song, Sophia Gunn, Stefano Monti, Gina M. Peloso, Ching-Ti Liu, Kathryn Lunetta, Paola Sebastiani

**Affiliations:** ^1^ Institute for Clinical Research and Health Policy Studies, Tufts Medical Center, Boston, MA, United States; ^2^ Department of Medicine, Tufts University School of Medicine, Boston, MA, United States; ^3^ The New York Genome Center, New York, NY, United States; ^4^ Section of Computational Biomedicine, Boston University School of Medicine, Boston, MA, United States; ^5^ Bioinformatics Program, Boston University, Boston, MA, United States; ^6^ Department of Biostatistics, Boston University School of Public Health, Boston, MA, United States; ^7^ Data Intensive Study Center, Tufts University, Medford, MA, United States

**Keywords:** Gaussian graphical models, corelated data, bootstrap, polygenic risk score, partial correlation

## Abstract

Gaussian Graphical Models (GGMs) are a type of network modeling that uses partial correlation rather than correlation for representing complex relationships among multiple variables. The advantage of using partial correlation is to show the relation between two variables after “adjusting” for the effects of other variables and leads to more parsimonious and interpretable models. There are well established procedures to build GGMs from a sample of independent and identical distributed observations. However, many studies include clustered and longitudinal data that result in correlated observations and ignoring this correlation among observations can lead to inflated Type I error. In this paper, we propose a cluster-based bootstrap algorithm to infer GGMs from correlated data. We use extensive simulations of correlated data from family-based studies to show that the proposed bootstrap method does not inflate the Type I error while retaining statistical power compared to alternative solutions when there are sufficient number of clusters. We apply our method to learn the Gaussian Graphic Model that represents complex relations between 47 Polygenic Risk Scores generated using genome-wide genotype data from the Long Life Family Study. By comparing it to the conventional methods that ignore within-cluster correlation, we show that our method controls the Type I error well without power loss.

## Introduction

One goal of biomedical research is to understand the network of complex relationships between biological variables and other factors to improve disease diagnosis and prognosis, and to identify drug targets (Vamathevan et al., 2019). The challenge of these analyses is the integration of the effect of multiple variables on more than one outcome of interest, simultaneously, and network modeling is a popular approach to address this task. Correlation networks are often used to model pairwise correlation, and, for example, weighted gene co-expression network analysis (WGCNA) is a popular solution to summarize the effects of multiple molecular features. Gaussian graphical models (GGMs) are a specific type of network modeling that use partial correlation rather than correlation to describe relations between may variables (Becker et al., 2023; Langfelder and Horvath, 2008). The advantage of GGMs is that they show the relation between two variables after “adjusting” for the effects of other variables and are therefore more parsimonious and interpretable. However, the calculation of the partial correlation typically assumes that all the variables are normally distributed (Markowetz and Spang, 2007).

The conventional method for learning a GGM is to perform hypothesis testing of the partial correlations that are derived from the normalized inverse of the variance-covariance matrix of the variables of interest (Whittaker, 2009). This approach roots in the assumptions that the variables follow a multivariate normal distribution, and the sample data consist of independent and identically distributed observations. The assumption of independent observations is violated whenever there is cluster sampling, for example, in family-based studies and several solutions have been proposed to learn GGMs from correlated data. Talluri and Shete adapted the Lasso-penalized maximum likelihood estimator of the precision matrix by incorporating the kinship matrix to account for the correlations introduced by family data (Talluri and Shete, 2014). However, this method requires prior knowledge of the variables’ heritability, which is not always available. Riberiro and Soler further leveraged the properties of family data for learning GGMs that are decomposed into the genetic and environmental networks (Ribeiro and Maria Pavan Soler, 2020). This approach is particularly useful if the goal is to distinguish between genetic and non-genetic contributions to the associations between variables in the model. However, the estimation and inference steps of the partial correlations are time-consuming due to large matrices decompositions and operations. Moreover, both approaches rely on the correct specification of the correlation structure underlying the data and are applicable only within family data framework.

In this work, we propose a cluster-based bootstrap algorithm to learn a GGM from correlated data. This method adapts the family-based bootstrap introduced by Borecki and Province (2008) to test the significance of the partial correlations between the variables and does not need knowledge of the correlations between the observations but only the cluster composition of the data. In addition, this approach is not limited to family-based data. Compared to regression-based methods that are challenged by the complexity of the search for a GGM, the computational complexity of this method remains polynomial. We show through a comprehensive simulation study that our algorithm controls the Type I error well, while retaining good statistical power. We also apply our method in a real-world example to show the impact of ignoring correlated data when building a GGM.

## Materials and methods

### Methods for learning Gaussian Graphic Models from independent observations

A Gaussian Graphic Model (GGM) is a statistical model that represents properties of marginal and conditional independences of a multivariate Gaussian distribution using an undirected Markov graph (Lauritzen, 1996; Whittaker, 2009). The key rule of an undirected Markov graph is that two variables are conditionally independent given all the other variables in the graph if they are not connected by an edge. Let $Y={\left(Y_{1},Y_{2},Y_{3},...,Y_{p}\right)}^{T}$ be a p -dimensional random vector with a multivariate normal distribution with mean vector μ and variance-covariance matrix Σ :

$$Y=\left(\begin{array}{c} Y_{1}\\ \vdots \\ Y_{p} \end{array}\right)∼MVN\left(\mu ,\Sigma \right)$$

Let G denote the associated undirected Markov graph from the set $\left(V,E\right)$ where $V=\left\{1,2,\ldots ,p\right\}$ is the vertex set corresponding to the univariate components Y , and the edge set $E=\{E_{i,j}:i,j\in V,i\neq \left.j\right\}$ describes the conditional dependency of random variables in Y (Kolaczyk, 2009). The strength of the conditional dependency of $Y_{i}$ and $Y_{j}$ after adjusting for all the other variables in Y is measured by the partial correlation $\rho_{ij}$ that is defined as:

$$\rho_{ij}=\frac{-k_{ij}}{\sqrt{k_{ii}k_{jj}}}\;\left(i\neq j\right)$$

where $k_{ij}$ is the ${\left(i,j\right)}^{th}$ entry of the precision matrix $K=\Sigma^{-1}$ (Whittaker, 2009). An edge exists between two vertices if the partial correlation between the two Gaussian random variables is not 0, i.e.,

$$E_{i,j}=1:\rho_{ij}\neq 0\;\left(i\neq j\right)$$

**FIGURE 1:**
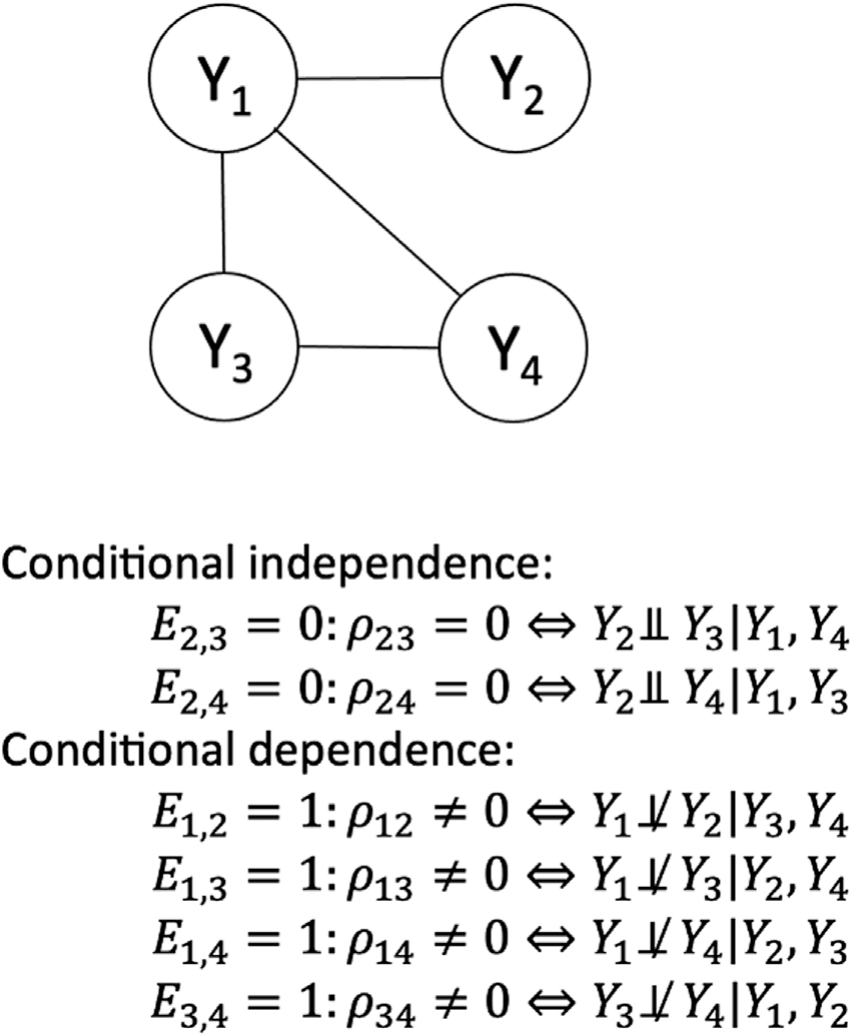
Example of a Gaussian graphical model with 4 vertices and 4 edges. This is a graph associated with vector $Y={\left(Y_{1},Y_{2},Y_{3},Y_{4}\right)}^{T}$ . Each vertex represents a variable, and the edges represents the conditional dependencies between each pair of variables given the rest of variables.

Figure 1 presents an illustrative GGM depicting the partial correlation network of the four-dimension vector $Y={\left(Y_{1},Y_{2},Y_{3},Y_{4}\right)}^{T}$ . The Markov graph shows that $Y_{2}$ is independent of $Y_{3}$ and $Y_{4}$ conditionally on $Y_{1}$ , and this relationship is represented by the missing edges $E_{2,3}=0,\rho_{23}=0$ and $E_{2,4}=0:\rho_{24}=0$ . The variables $Y_{1}$ and $Y_{3}$ are dependent on each other when conditioned on $Y_{2}$ and $Y_{4}$ , which can be described by the existing edge $E_{1,3}=1:\rho_{13}\neq 0$ .

We test the null hypothesis of conditional independence of $Y_{i}$ and $Y_{j}$ given all the other variables, say ${H_{0}:\rho }_{ij}=0$ against the alternative hypothesis ${H_{1}:\rho }_{ij}\neq 0$ , using the Fisher’s Z -transformation test:

$$R_{i,j}=\frac{1}{2}\log\left(\frac{1+{\hat{\rho }}_{ij}}{1-{\hat{\rho }}_{ij}}\right)$$

where ${\hat{\rho }}_{ij}$ is the estimate of $\rho_{ij}$ from a sample with sample size N (Ronald Aylmer, 1921). It is well known that the distribution of the statistic under the null hypothesis ${H_{0}:\rho }_{ij}=0$ can be approximated by

$$R_{i,j}\;∼\;N\left(0,\frac{1}{N-p-3}\right)$$

when the sample size N is large and the observations are independent and identically distributed (Fisher, 1924).

### Clustered data issues

The assumption of independent and identically distributed observations is violated in studies with correlated data such as cluster-based sampling or family-based recruitment (Laird, 2004). Subjects within a cluster are correlated due to shared environment components or sharing of genetic factors in family-based studies (Wojczynski et al., 2022). Failure to account for these correlations can lead to false positive results (Cannon et al., 2001).

In the analysis of cluster data, often investigators assume an exchangeable covariance structure, where the correlation of pairs of subjects in the same cluster is constant. Family data is a special type of clustered data in which each family is a cluster unit. The correlation structure of family data can be more complex with correlations between pairs of subjects that depend on their family relationship and shared environment. In genetic studies, the variance of a trait Y is commonly decomposed into two components: the environmental and the genetic components (Almasy and Blangero, 1998; Amos, 1994). Denote by $y_{mk}$ the observation of a variable Y in the $k^{th}$ individual from the $m^{th}$ family. The effect of the two components of variance is usually parameterized as

$$y_{mk}=\mu +{\;e}_{mk}+{\;g}_{mk},$$

where ${\;e}_{mk}\;∼\;N\left(0,\sigma_{e}^{2}\right)$ denotes the environmental component, ${\;g}_{mk}\;∼\;N\left(0,\sigma_{g}^{2}\right)$ denotes the genetic component, and they are mutually independent. The environmental components ${\;e}_{mk}$ are assumed to be independent for any m,k while the genetical components ${\;g}_{mk}$ are independent between different families and dependent within families. Therefore, for any two subjects from different families, the observations $y_{mk}$ and $y_{lh}$ are independent but the observations of two subjects from the same family m , say $y_{mk}$ and $y_{mh}$ are correlated

$$cov\left(y_{mk},y_{mh}\right)=2^{-d}\sigma_{g}^{2}$$

The parameter d describes the degree of relatedness between the two individuals. The coefficient $2^{-d}$ , also known as the family relatedness coefficient, ranges from 0 to 1. A value of 0 indicates that the individuals are independent, while a value of 1 signifies that they are genetically identical, as in the case of monozygotic twins. The generating model for one trait with sample data $Y_{N\times 1}$ can be written in matrix form as

Y=μ+E+G

where the vector of the environmental components is parameterized as $E∼MVN\left(0,\sigma_{e}^{2}I_{N}\right)$ , with $I_{N}$ denoting the N×N identity matrix, and the vector of the genetic components is parameterized as $G∼MVN\left(0,\sigma_{g}^{2}\Phi \right)$ , with Φ denoting a N×N block diagonal matrix called the relatedness matrix. The h,k element of Φ represent the relatedness between individuals h,k and are 0 when individuals h,k are from different families, and they are $2^{-d}$ when individuals h,k are a d -*t*ℎ degree relative pair (Lange, 2022).

We next extend the parameterization for the multivariable case in which we assume to have p variables. We denote by Y the Np×1 vector of the stacked sample data:

$$Y=\left(\begin{array}{c} Y_{1}\\ \vdots \\ Y_{p} \end{array}\right)$$

And we model Y as

Y=μ+E+G

where μ is the Np×1 vector of means of the p variables, and the Np×1 vectors of environmental and genetic components as $E∼MVN\left(0,I_{N}⊗\Sigma_{e}\right)$ , and $G∼MVN\left(0,\Phi ⊗\Sigma_{g}\right)$ , where the symbol ⊗ denotes the Kronecker product. Our goal is to learn the GGM by leveraging the precision matrix $K=\Sigma^{-1}$ , where Σ represents the variance matrix in an independent data setting. In the Supplementary Material, we demonstrate that when extending to a family data setting, $\Sigma =\Sigma_{e}+\Sigma_{g}$ . However, directly evaluating Σ , $\Sigma_{e}$ and $\Sigma_{g}$ in the presence of correlated data is computationally complex. For computational feasibility, we wish to use the statistic $R_{i,j}$ but the challenge of correlated data is that the distribution of the statistic $R_{i,j}$ is unknown. We therefore use the cluster-based bootstrap method to address this issue.

### Bootstrap algorithm on clustered data

The bootstrap method, introduced by Efron (1979), is a widely used resampling technique for statistical inference and hypothesis testing. It involves resampling the data with replacement and then using these samples to estimate the distribution of a statistic of interest. Sherma and Cessie suggested that the bootstrap method could also be used to address issues with correlated data by resampling clusters instead of individuals (Sherman and le Cessie, 1997), and Borecki and Province introduced a family-based bootstrap approach in which the sample units are families and familial relations are ignored in the estimation phase (Borecki and Province, 2008).

Here we propose a generalization of the family-based bootstrap algorithm introduced by Boreki and Province to learn GGMs that account for correlated observations. The steps of the proposed cluster-based bootstrap algorithm are as follows:

Step 1 For t=1,2,…,T where T represents the number of bootstrap resampling

i. Draw c% of clusters with replacement from the cluster data, e.g., draw $\frac{cM}{100}$ families with replacement, where M is the number of families in the data set;
ii. Calculate the sampling variance-covariance matrix $S^{\left(t\right)}$ using the data resampled in step i. and calculate the partial correlation matrix $P^{\left(t\right)}$ as:

$$P^{\left(t\right)}=-{D^{\left(t\right)}}^{-\frac{1}{2}}{S^{\left(t\right)}\;}^{-1}{D^{\left(t\right)}}^{-\frac{1}{2}}$$ (4)

where $D^{\left(t\right)}$ is a diagonal matrix from the diagonal elements of ${S^{\left(t\right)}\;}^{-1}$ ;

iii. For each pair $Y_{i}$ and $Y_{j}$ , calculate the Fisher’s transformation statistics ${R_{i,j}}^{\left(t\right)}$ :

$${R_{i,j}}^{\left(t\right)}=\frac{1}{2}\log\left(\frac{1+{\rho_{ij}}^{\left(t\right)}}{1-{\rho_{ij}}^{\left(t\right)}}\right)$$

where ${\rho_{ij}}^{\left(t\right)}$ is the ${\left(i,j\right)}^{th}$ entry of $P^{\left(t\right)}$ . Calculate the standard deviation of ${R_{i,j}}^{\left(t\right)}$ as $1/\sqrt{\left(N^{\left(t\right)}-p-3\right)}$ where $N^{\left(t\right)}$ is the overall sample size at the $t^{th}$ iteration. Calculate the variable ${{R_{i,j}}^{*}}^{\left(t\right)}$ as

$${{R_{i,j}}^{*}}^{\left(t\right)}=\sqrt{\left(N^{\left(t\right)}-p-3\right)}{R_{i,j}}^{\left(t\right)}$$

Step 2 Calculate the bootstrap estimate of the standard deviation of ${R_{i,j}}^{*}$ as:

$$\hat{se\left({R_{i,j}}^{*}\right)}=\sqrt{{\frac{1}{T-1}\sum_{t=1}^{T}\;\left({{R_{i,j}}^{*}}^{\left(t\right)}-\frac{1}{T}\sum_{t=1}^{T}{{R_{i,j}}^{*}}^{\left(t\right)}\right)}^{2}}$$

where

$${R_{i,j}}^{*}=\sqrt{\left(N-p-3\right)}R_{i,j}$$

Here $R_{i,j}$ is the Fisher’s transformation statistic calculated from the sampling variance-covariance matrix S from the original dataset. We can construct a standard normal test statistic as

$$Z_{i,j}=\frac{{R_{i,j}}^{*}}{\hat{se\left({R_{i,j}}^{*}\right)}}$$

Step 3 Test the null hypothesis at level α if $\left|Z_{i,j}\left|>\;\right.Z_{\alpha /2}\right.$ .

### Simulation settings

**FIGURE 2:**
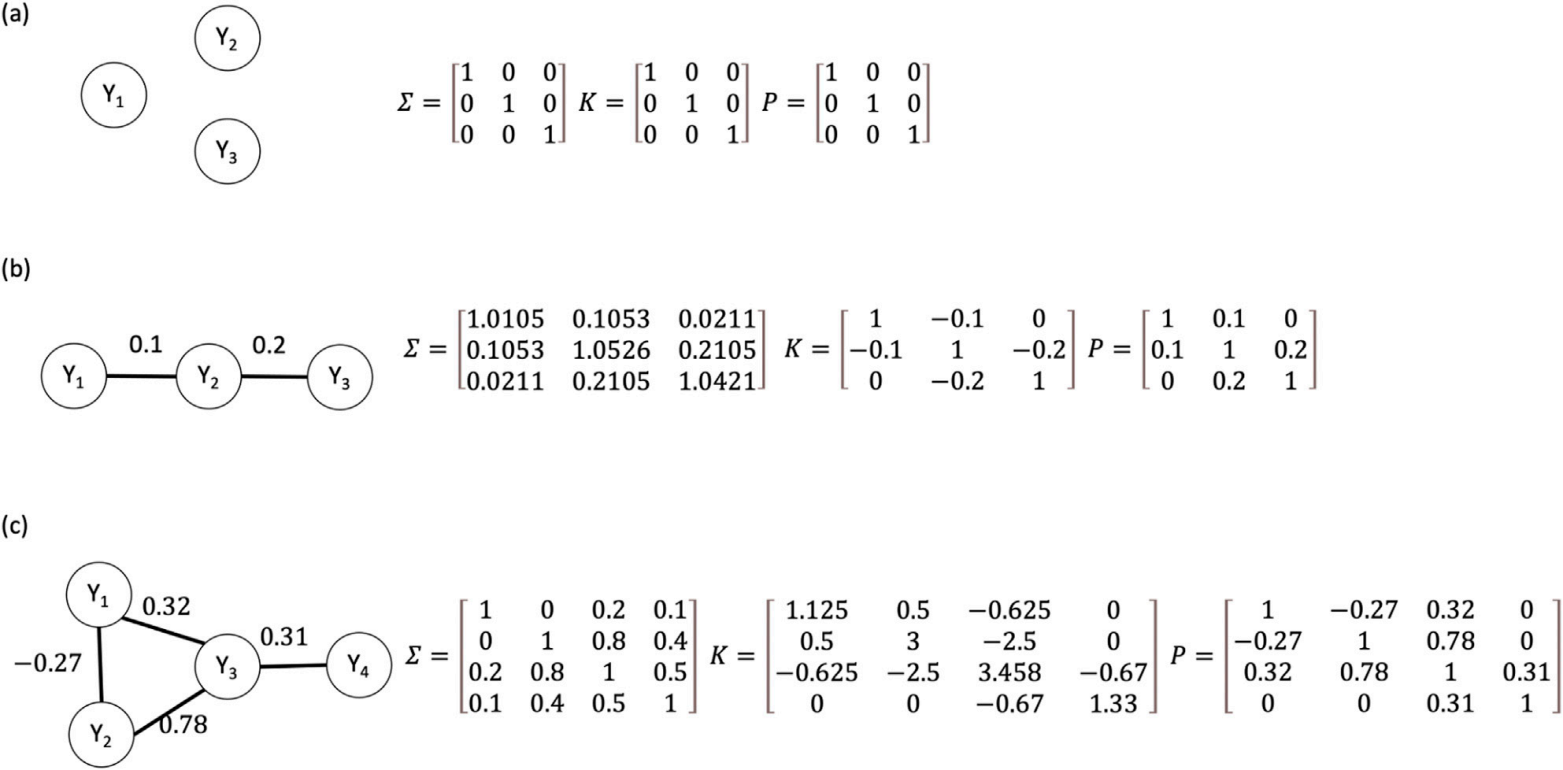
Graphical representation of the three simulation Markov models used in simulation studies. **(a)** The independence graph **(b)** the chain graph **(c)** the triangle tail graph. Each graph is accompanied by Σ , the variance-covariance matrix; K , the precision matrix; and P , the partial correlation matrix highlighting the edges.

In the simulation study, we used the method described in the appendix to simulate a multivariable data set with related individuals. We simulated a mixed family structure where half of the families consisted of two parents and one offspring, while the other half consisted of two parents and five offspring. We simulated observations assuming three different numbers of families: 40, 120, and 360 families, resulting in sample sizes of N= 200, 600, and 1800, respectively. We varied the heritability values $h^{2}$ (defined as the ratio between genetic and total variance) from 0 to 0.95 with increments of 0.125, 0.25, 0.5, and 0.75. We also generated 1,000 datasets for each combination of sample size and heritability. We simulated data from three different GGMs with Markov graphs depicted in Figure 2. The first model included three variables that were marginally independent of each other, so that the Markov graph did not include any edge (Figure 2a). The second model was represented by a chain graph describing two variables $Y_{1}$ and $Y_{3}$ conditionally independent given $Y_{2}$ (Figure 2b). The third model was a triangle tail graph that described ${\;Y}_{1}$ , $Y_{2}$ and $Y_{3}$ connected to each other and $Y_{4}$ is independent of $Y_{1}$ and $Y_{2}$ conditional on $Y_{3}$ (Figure 2c). The p×p variance covariance matrix Σ , precision matrix K , and corresponding partial correlation matrix P for each graph are displayed in Figure 2. Without loss of generality, we set the mean µ of each variable to be 0. In addition to these three basic graphs, we simulated a complex network reflecting the partial correlation matrix of the first 30 Polygenic Risk Scores (PRS) from our real data application. In this scenario, partial correlations less than 0.01 were set to zero. To accommodate the increased number of nodes, we included an additional simulation setting with 1,080 families ( N=5400 ).

For each scenario, we learned the GGM structure by using:

• The Fisher’s Z -transformation test for partial correlations ignoring the family-based design.
• The naïve bootstrap algorithm, where we resampled 50 datasets, and each time sampling 100% individuals with replacement.
• Our proposed cluster-based bootstrap algorithm, in which we resampled 50 and 200 datasets, and for each resample, we drew 50% , 75% and 100% of families with replacement.

We used a level of statistical significance α = 0.05 for the Hypothesis test of each $\rho_{i,j}$ and estimated the false positive rate (FPR) by the proportion of incorrect edges found to be significant. The statistical power was evaluated using this algorithm: when inflation in FPR was presented in the conditionally independent variable pairs in one graph, we corrected the level of significance α by the mean inflation rate, which is the average of the FPR divided by α , and conducted a new hypothesis test at $\alpha^{*}$ :

$$\alpha^{*}=\frac{0.05}{\bar{FPR}/\alpha }$$

We repeated this process until $\alpha^{*}=0.05$ and then used this adjusted significance level to estimate the power as the proportion of edges found between the variable pairs that were connected in the data model.

### Study cohort

The Long Life Family Study (LLFS) is a family-based study of healthy aging and longevity that recruited over 5,000 family members in approximately 550 families selected for familiar longevity. Participants were enrolled at three American field centers in Boston, Pittsburgh, and New York, along with a European field center based in Denmark (Sebastiani et al., 2009; Wojczynski et al., 2022). Socio-demographic, medical history data, current medications, physical and cognitive function data, and blood samples were collected via in-person visits and phone questionnaires for all subjects at the time of enrollment and during follow-ups (Elo et al., 2013; Newman et al., 2011). Genome-wide genotype data were generated at the Center for Inherited Disease Research (CIDR) using the Illumina Omni 2.5 platform, and genotype calls were cleaned as described in (Bae et al., 2013). The genotype data were imputed with Michigan Imputation Server to the HRC panel (version r1.1 2016) (Das et al., 2016). Genome-wide genotype data are available from dbGaP (dbGaP Study Accession: phs000397.v1.p1). We augmented the genetic data in the LLFS using approximately 3,500 samples that we used as controls in other studies of longevity (Bae et al., 2013). The genotype data are accessible at http://www.illumina.com/documents/icontroldb/document_purpose.pdf. We used these genetic data to calculate the Polygenic Risk Scores (PRS) for 54 health outcomes summarized in Supplementary Table S3. These PRS were calculated as the weighted sum of individual’s genetic variants associated with the corresponding outcome and all the details are described in reference (Gunn et al., 2022).

### Implementation and code availability

The code used in this study is available upon request and can also be accessed on GitHub at: https://github.com/QM-DS-Tufts-Medical-Center/GGM-network-Bootstrap.git.

## Results

### The simulation studies demonstrate that the bootstrap-based approach controls the type I error without losing power

**FIGURE 3:**
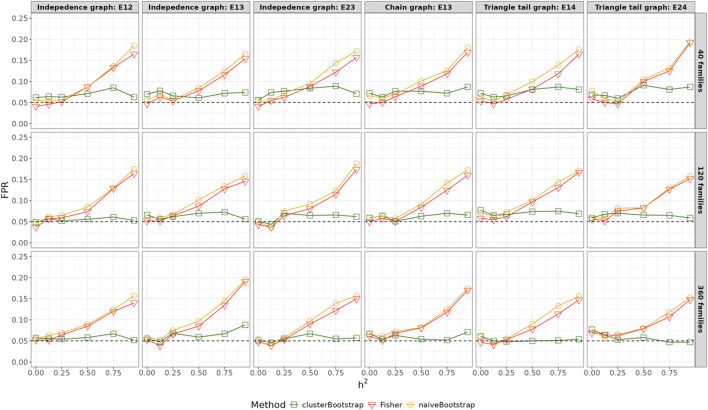
False positive rates across varying heritability for all edges with $E_{i,j}=0$ in the first three graphs. The dashed line represents the constant significance level α=0.05 for each hypothesis test. The FPR of Fisher’s method (Fisher) is indicated by red triangles, and the FPR of naïve Bootstrap (naïveBootstrap) is indicated by yellow circle, both showing an increasing inflation as heritability increases. In contrast, the FPR of the proposed Bootstrap method (clusterBootstrap), represented by green square, remains constant regardless of heritability.

**FIGURE 4:**
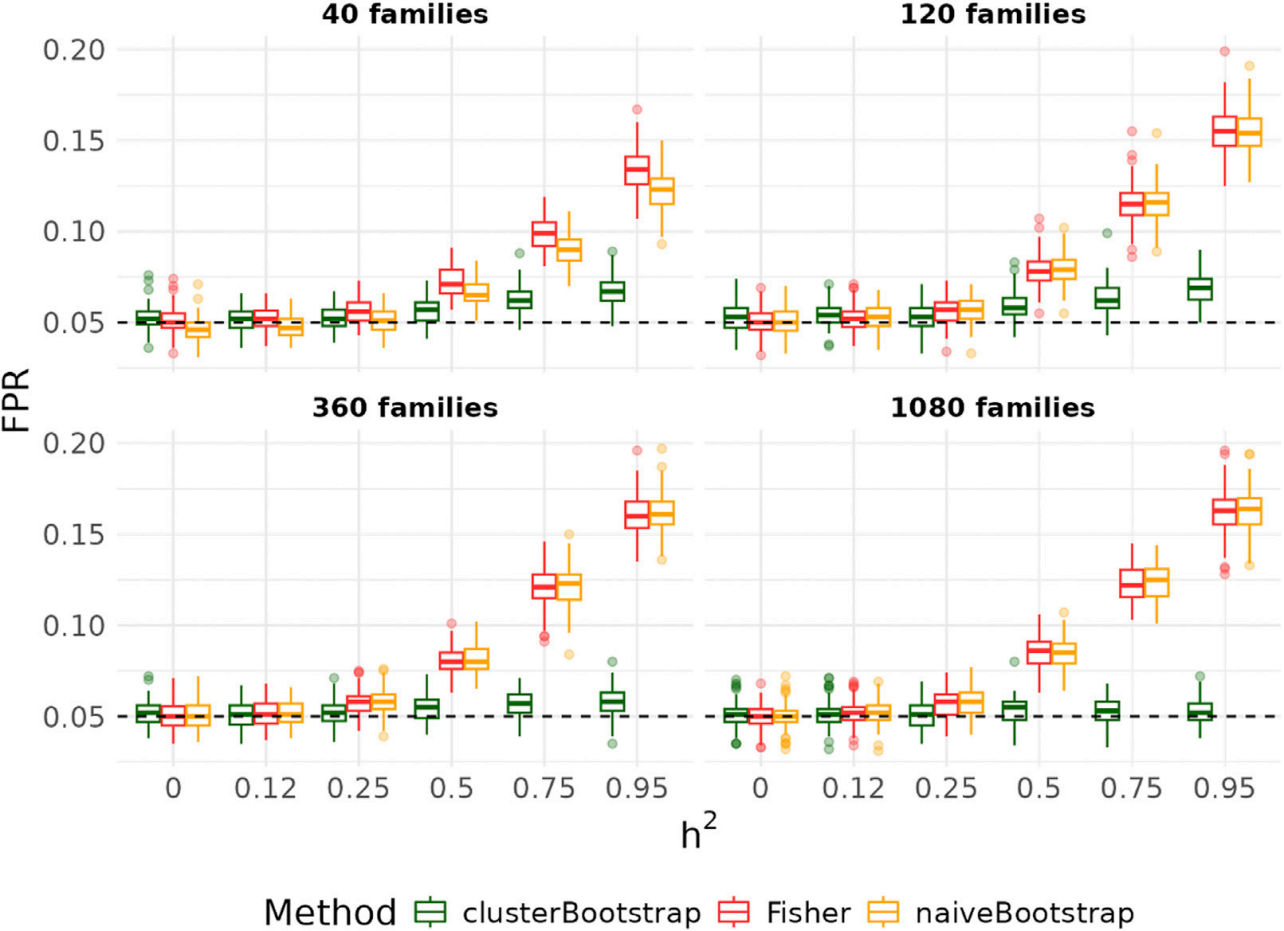
False positive rates across varying heritability for all edges with $E_{i,j}=0$ in the simulated PRS graph. The dashed line represents the constant significance level α=0.05 for each hypothesis test. The FPR of Fisher’s method (Fisher) is indicated by red, and the FPR of naïve Bootstrap (naïveBootstrap) is indicated by yellow, both showing an increasing inflation as heritability increases. In contrast, the FPR of the proposed Bootstrap method (clusterBootstrap), represented by green, remains constant regardless of heritability when number of clusters are large.

Figures 3, 4 summarizes the results of the FPR for different scenarios and methods. The FPR of the Fisher’s test and the naïve bootstrap algorithm that ignores the family structure increased across all three scenarios as the heritability levels increased, and the inflation rates increased by 2 to 4-fold when the heritability exceeds 0.5. When the number of families was small ( M=40 ), the cluster-based bootstrap algorithms exhibited an inflated FPR of approximately 1.5-fold across varying heritability levels. However, as the number of families increased to 120 and 360, the cluster-based bootstrap algorithms consistently maintained the FPR at 0.05 across all heritability levels in the simple graph settings with 3–4 nodes (Figure 3; Supplementary Figure S1; Supplementary Table S1). In the more complex PRS graph scenario (Figure 4), which includes 30 nodes, FPR inflation is observed in the proposed cluster-based bootstrap method when the number of clusters is 40 or 120 and heritability exceeds 0.25. As the number of clusters increases, the FPR inflation diminishes for the proposed method. In contrast, both the naïve bootstrap and Fisher’s test consistently show inflated FPRs across all settings as long as the heritability exceeds 0.12 (Supplementary Table S2).

**FIGURE 5:**
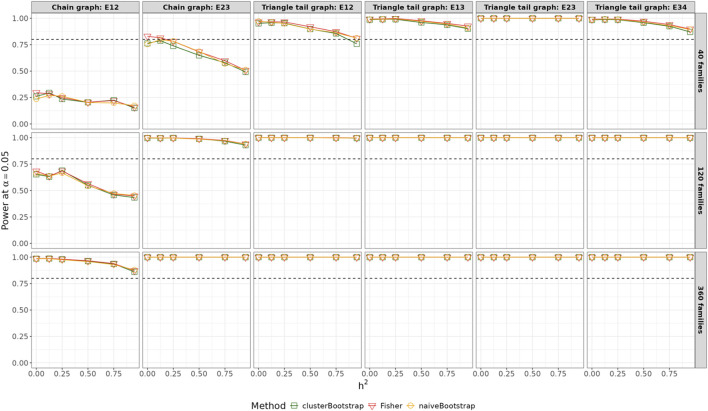
Power across varying heritability for all edges with $E_{i,j}=1$ in the first three graphs. Power is evaluated at the adjusted significance level $\alpha^{*}=0.05$ , as described in the power evaluation section. The dashed line represents the constant power at 0.8. Power decreases for all three methods as heritability increases. The power of the proposed Bootstrap method (clusterBootstrap), represented by green square, is comparable to both the Fisher’s method (Fisher) and the naïve Bootstrap method (naiveBootstrap).

**FIGURE 6:**
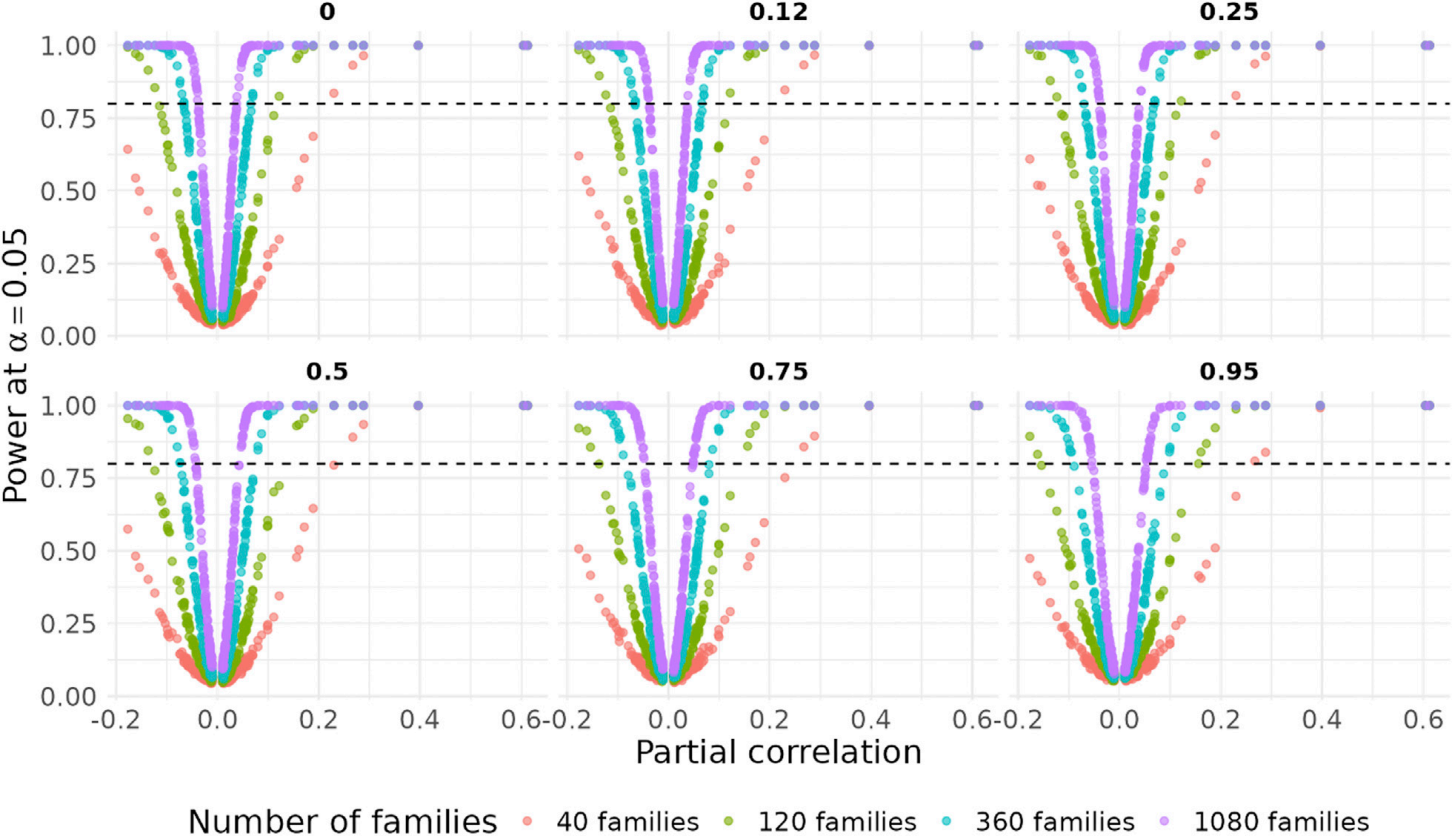
Power across varying heritability for all edges with $E_{i,j}=1$ in the simulated PRS graph using proposed Bootstrap method. Power is evaluated as the proportion of edges identified among 1,000 simulated datasets. The dashed line represents the constant power at 0.8. Power decreases as heritability increases.

Figure 5 summarizes the statistical power of detecting true edges using the Fisher’s test, the naïve bootstrap method and the cluster-based bootstrap algorithm as a function of the heritability in the simple graph settings. As heritability increased, the statistical power decreased particularly with small sample sizes and partial correlations between variables close to 0. However, when the number of families exceeded 360, the power remained consistently above 0.8, irrespective of the heritability values ranging from 0 to 0.95. For smaller family sizes ( M=40 ), a power above 0.8 was achieved if the magnitude of the partial correlations exceeded 0.3, regardless of heritability. The bootstrap algorithm that sampled 100% of families showed comparable power to the Fisher’s test and the naïve bootstrap method, whereas sampling 75% of families resulted in lower power due to the reduced sample size (Supplementary Figure S2). In the simulated PRS graph setting, we further examined how power varies with the magnitude of the partial correlations (Figure 6). Power decreases with increasing heritability but increases with stronger partial correlations. Edges with partial correlations greater than 0.3 maintain power above 0.8 even when the number of clusters is small.

Comparing the proposed cluster-based bootstrap algorithm that samples 100% of the clusters with the Fisher’s test and naïve bootstrap method, we observed comparable power across all scenarios and well-controlled the Type I error rate when the number of clusters is sufficiently large. Our results indicated that bootstrapping 50/200 datasets with 100% families resampled yielded the best performance in terms of both power and FPR. However, reducing the proportion of families resampled leads to a decrease in power.

### The GGM of polygenic risk scores highlight groups of traits with correlated genetic risks

We applied this new algorithm to characterize the mutual correlations between 47 polygenic risk scores in the LLFS (Gunn et al., 2022; Wojczynski et al., 2022). Polygenic Risk Scores (PRS) for 54 health outcomes using genetic data of 8,190 samples were calculated as described in the methods (Gunn et al., 2022). These PRS reflect the relative genetic risk of developing the outcome in carriers of combinations of risk variants compared to non-carriers. These 54 outcomes include age-related diseases such as Alzheimer’s disease, coronary artery disease, and a variety of other traits related to mental health (e.g., bipolar disorder), and general physiological characteristics as listed in Supplementary Table S3. We further removed two PRS with very skewed distribution (Supplementary Figure S3) and an additional five PRS that had several potential outliers that lie 4 standard deviations away from the means (Supplementary Figure S4). We learned the partial correlation networks of the remaining 47 PRS using three methods: Fisher’s transformation test with independent subsets of the data yielding a sample size of N=4193 , Fisher’s test on all data ignoring the correlation within families ( N=8190 ), and the proposed cluster-based bootstrap algorithm ( N=8190 ). In the first method, we generated independent subsets by randomly sampling one subject per family. In the second method, we used the Fisher’s test to all data ignoring the family-based correlation. With the bootstrap algorithm, we sampled 1,000 datasets with 100% of families sampled each sampling. We applied Bonferroni correction to control the family-wise error rate (FWER) to be 0.05.

**FIGURE 7:**
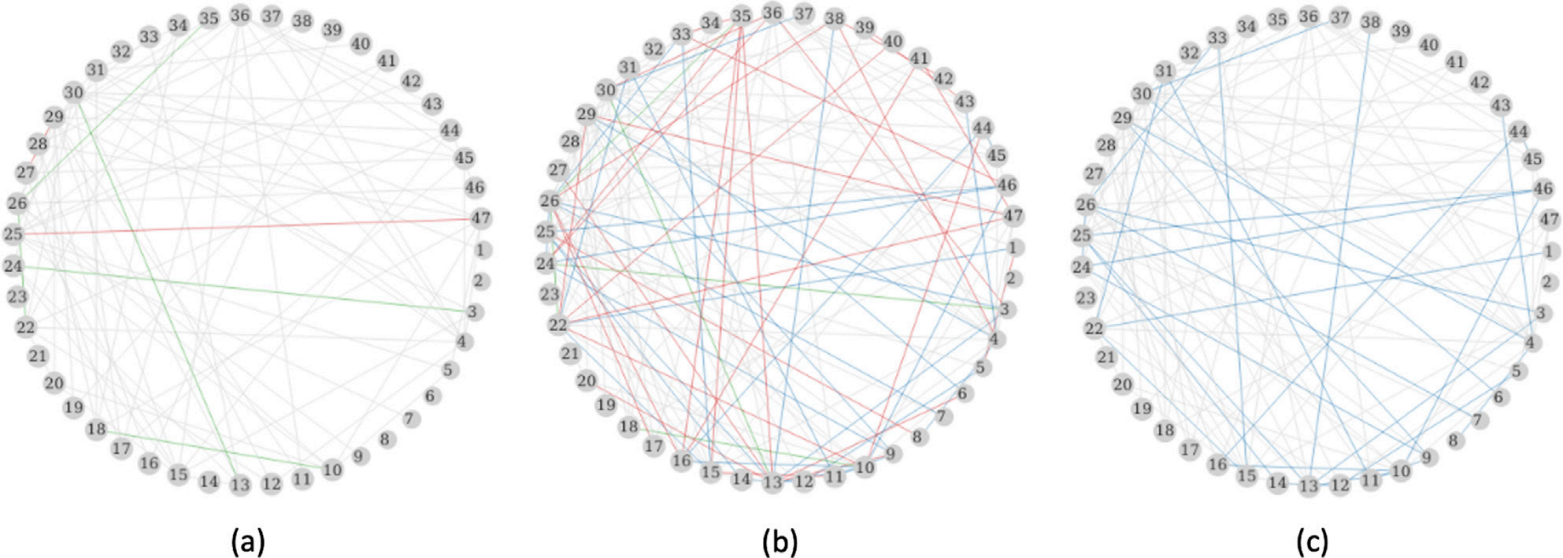
PRS networks inferred with Bonferroni correction. **(a)** Fisher’s test on sampled iid data, **(b)** Fisher’s test on all data, and **(c)** Bootstrap resampling 1,000 datasets and 100% families. Grey edges are edges identified in all three approaches; green edges are intersections by **(a)** and **(b)**; blue edges are intersections by **(b)** and **(c)**; red edges are identified only by the corresponding approach. The numbers in each circle is a PRS as listed in Supplementary Table S1.

Figure 7 displays the networks constructed using the three methods. The Fisher’s Z -transformation test identified 85 edges (7.86% of the total 1,081 possible edges) using data of 4,193 independent subjects, while using the Fisher’s Z -transformation test ignoring the correlations within families identified 143 (13.2%) edges using data from 8,190 subjects. The cluster-based bootstrap method applied to the data set of 8,190 subjects identified a total of 108 (9.99%) edges (Table 1; Supplementary Figure S5). As expected, this number was between the previous two methods since the analysis of the independent observations used a sample size reduced by almost 50% and was less powerful, while the method that analyzed all the data ignoring correlations within families likely introduced false positive edges. Table 1 and Figure 6 showed that the three algorithms identified 78 edges in common. The cluster-based bootstrap algorithm identified an additional 30 edges that were also identified with the Fisher’s test applied to all samples (Supplementary Table S4). However, the latter method identified an additional 30 edges that were not identified by either the Fisher’s Z -transformation test on independent sample nor the bootstrap algorithm (Supplementary Table S5). In the PRS network learnt with the bootstrap method, 43 PRS connected to each other and formed a single large cluster. The PRS for intelligence had the highest degree and it connected to 14 other PRSs. These connections included traits such as birth weight, height, educational attainment, cognitive performance, and parental extreme longevity.

**TABLE 1:** Comparison of common edges inferred by the three methods.

Edges by Fisher’s (iid sample)	Edges by Fisher’s (all sample)	Edges by bootstrap	Counts
0	0	0	936
0	1	0	30
1	0	0	2
1	1	0	5
0	0	1	0
0	1	1	30
1	0	1	0
1	1	1	78

This table compares the number of common edges identified between pairs of PRS networks using the three different methods. An edge value of 0 indicates no connection between two PRS, while an edge value of 1 indicates a connection. The table shows that 78 pairs of PRS are identified as connected by all three methods, whereas 936 pairs are identified as not connected by all three methods.

### Evaluation of computation time

**FIGURE 8:**
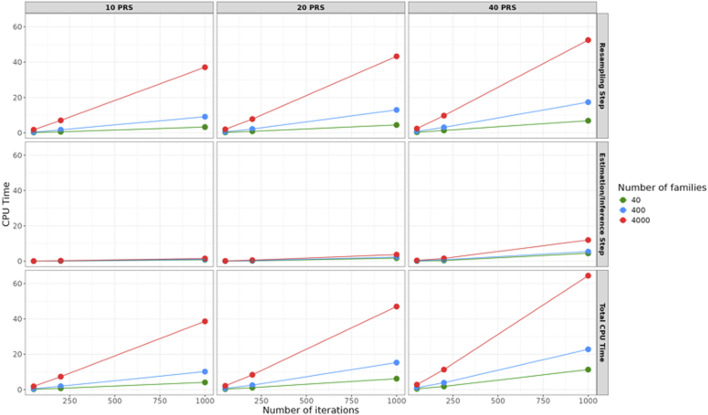
The CPU time (in seconds) for the Bootstrap algorithm. The CPU time increases with the number of families, PRS, and iterations. The figure shows results for 50, 200, and 1,000 iterations. According to the simulation study, more than 50 iterations are generally sufficient. For large numbers of families, selecting 50 to 200 iterations can keep the total CPU time within 15 s.

We evaluated the computation time of the cluster-based bootstrap algorithm by calculating the CPU time of the resampling steps and the inference steps. We ran the evaluation using a single computer node with 1 core and R version 4.1.1. We sampled 40, 400, and 4,000 families from the LLFS data, and sampled 10, 20, 40 PRS. For each scenario, we obtained the computational time for 50, 200 and 1,000 iterations with 100% of the families resampled each iteration. The algorithm finished in 65 s in the scenario with 40 PRS, 4,000 families and 1,000 iterations as shown in Figure 8. Notably, the resampling step took 52 s that makes up to 81% of the total CPU time.

## Discussion

In this study, we introduced a novel cluster-based bootstrap algorithm for learning partial correlation networks from correlated data. We showed in simulated data that this algorithm effectively controls the FPR while maintaining comparable power performance to conventional methods. Although we described the method in the context of family data, the cluster-based bootstrap algorithm can be directly applied to any correlated data setting without explicitly modeling the correlation structure, as it is required in (Ribeiro and Maria Pavan Soler, 2020).

To evaluate our approach, we conducted a comprehensive simulation study that highlighted strengths but also potential limits of this approach. The analysis showed that the algorithm controls the Type I error well without loss of power when both the number of families/clusters and the number of subjects large, such as more than 40, and many subjects. This is consistent with the well-known limitation that the bootstrap estimate of the standard error is not accurate when the number of clusters is small and the test statistics tends to be too liberal (Huang, 2018). This bias leads to a higher FPR regardless of the heritability. Another limitation arises when the sample sizes are smaller than the number of variables, as the test statistic is no longer well-defined. When the heritability of traits is less than 25%, the cluster-based bootstrap algorithm behaves similarly to the Fisher’s test and the naïve bootstrap method that ignores the correlation. However, in omics data analyses, most traits exhibit high heritability, typically exceeding 30%. In such cases, it would be optimal to use the cluster-based bootstrap algorithm if investigators have sufficient computational capacity.

The application of the approach to the PRS analysis shows that correcting for the within-family correlation reduced the number of edges from 143 to 108 and was more powerful than analyzing a subset of independent observations. Through the network analysis, we identified PRS that functioned as central nodes with multiple connections to other PRS. These central nodes include intelligence, ankylosing spondylitis, juvenile idiopathic arthritis, height, heel bone mineral density, and cognitive performance. Our cluster-based method preserved important connections, such as the edge between cognitive performance and FEV1 (Richards et al., 2005), that were missed by Fisher’s test applied to the independent subset. Some of the edges that were detected ignoring the correlation in the observations appeared to be false positive, for example, the edge between the PRS for intelligence and for FEV1. While our method effectively reduces false edges caused by correlated data, the resulting network remains highly connected and challenging to interpret. In future work, we will extend this method to learn sparse networks that yield more interpretable graphs.

The computational efficiency of our algorithm is a function of various factors, including the number of bootstrap iterations, the number of vertices, the number of families/clusters and total number of samples. The number of families impacts the resampling procedure’s runtime, while the number of nodes influences the calculation of partial correlation matrices. Furthermore, the cumulative effect of resampling and partial correlation calculations per iteration significantly contributes to the time needed for constructing Z-scores. A potential improvement to computational efficiency would involve a faster algorithm for calculating the inverse of the variance covariance matrix especially when the number of vertices are very large.

The learning strategy implemented through our proposed algorithms relies on testing multiple null hypotheses $\rho_{ij}=0$ against the alternative hypotheses $\rho_{ij}\neq 0$ . It is important to adjust the significance levels for these tests to control the family wise error rate. However, due to the non-independent nature of the performed tests, it is challenging to achieve precise adjustments (Ribeiro and Maria Pavan Soler, 2020). In the learning of the PRS networks, we applied the stringent Bonferroni correction to control the FWER without accounting for the effective numbers of tests, which could lead to overcorrecting as shown by Drton and Perlman (2007). In future work, we would like to introduce a better way to control the FWER. As an alternative to controlling the FWER, the FDR procedure by Liu (2013) is also a good solution that can be integrated into our algorithm.

This work has some limitations. For example, we conducted simulation studies using genetically independent traits. It is not straightforward to extend our simulations to genetically correlated traits since the variance-covariance matrix $var\left(Y\right)=\left(\Phi -I\right)⊗H\Sigma +I⊗\Sigma$ is not guaranteed to be positive semi-definite when H is not diagonal. However, the application to the PRS in LLFS showed that our bootstrap method works well even with some genetic correlations among traits. In fact, the heritability of PRS is very high as shown in Supplementary Table S6 and the PRS are genetically correlated since many of the outcomes shared common SNPs. In addition, we limited our simulation study to 2-generation families, but it will be interesting to expand this study to multi-generation families with a variety of relatedness patterns. Our simulation assumed no inbreeding and an additive genetic model, and some evaluation would be necessary to evaluate the validity of this approach to different genetic models and other types of correlated data. Finally, we did not include comparisons with Lasso-based methods that address specifically the issue of sparsity (Meinshausen and Bühlmann, 2010). This is an important topics that we will address in future work. We did not address the impact of skewed or heavy-tailed distributions, and we acknowledge that this remains an important issue that needs further investigation before our method can be applied to non-normally distributed data.

## Conclusion

By displaying conditional dependencies into patterns of edges in a network, GGMs offer a great statistical tool to represent intricate relationships within data in an intuitive manner and could be potentially very useful in the emerging field on multi-omics integration. However, the generation of GGMs from correlated data is a challenging task. We provided a simple method to derive a GGM from correlated data that is computationally efficient and appears to control the FPR without losing statistical power. This approach could increase the use of GGMs in observational study data that often, by design, generate correlated observations.

## Data Availability

The data analyzed in this study is subject to the following licenses/restrictions: The LLFS data are available from dbGaP (dbGaP Study Accession: phs000397.v1.p1). Requests to access the simulated datasets should be directed to zeyuan.song@tuftsmedicine.org.
