# Supplementary figures and images for: Learning Gaussian graphical models from correlated data
Source: Front Syst Biol. 2025 Jul 3;5:1589079. doi: 10.3389/fsysb.2025.1589079 (PMC12323441; doi:10.3389/fsysb.2025.1589079)

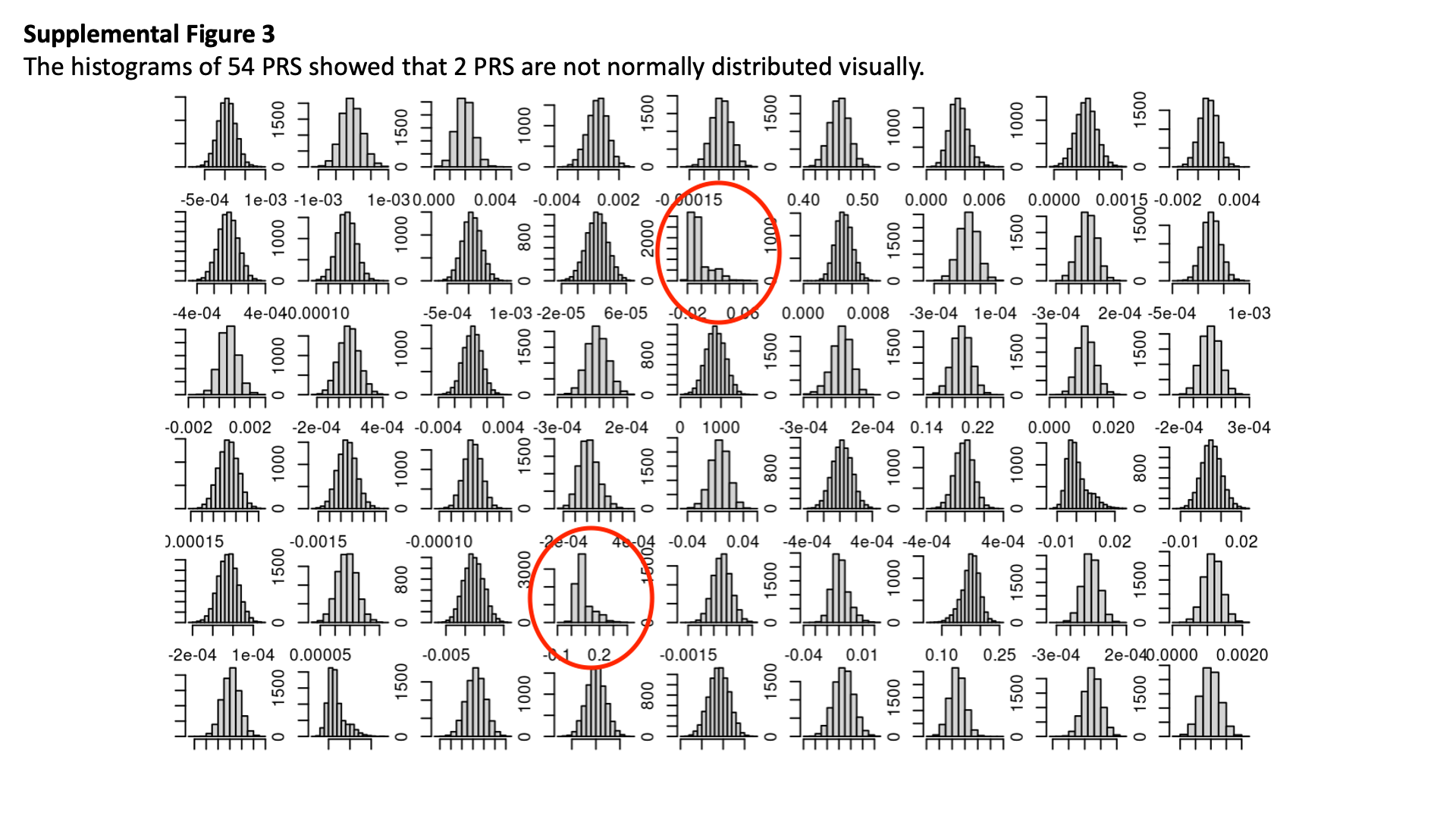

Supplement: Supplementary file 1 [file Image3.tiff]

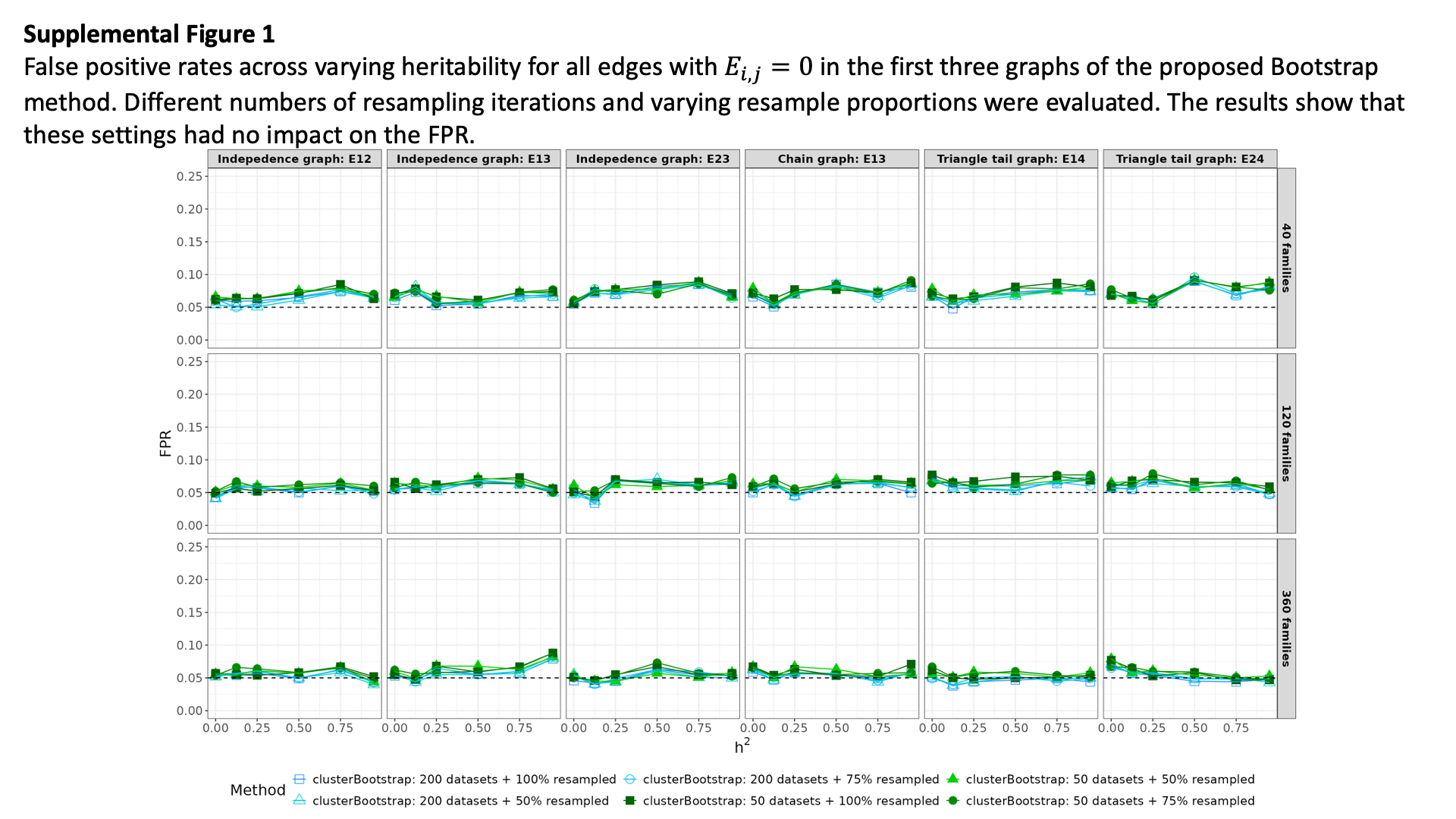

Supplement: Supplementary file 2 [file Image1.tiff]

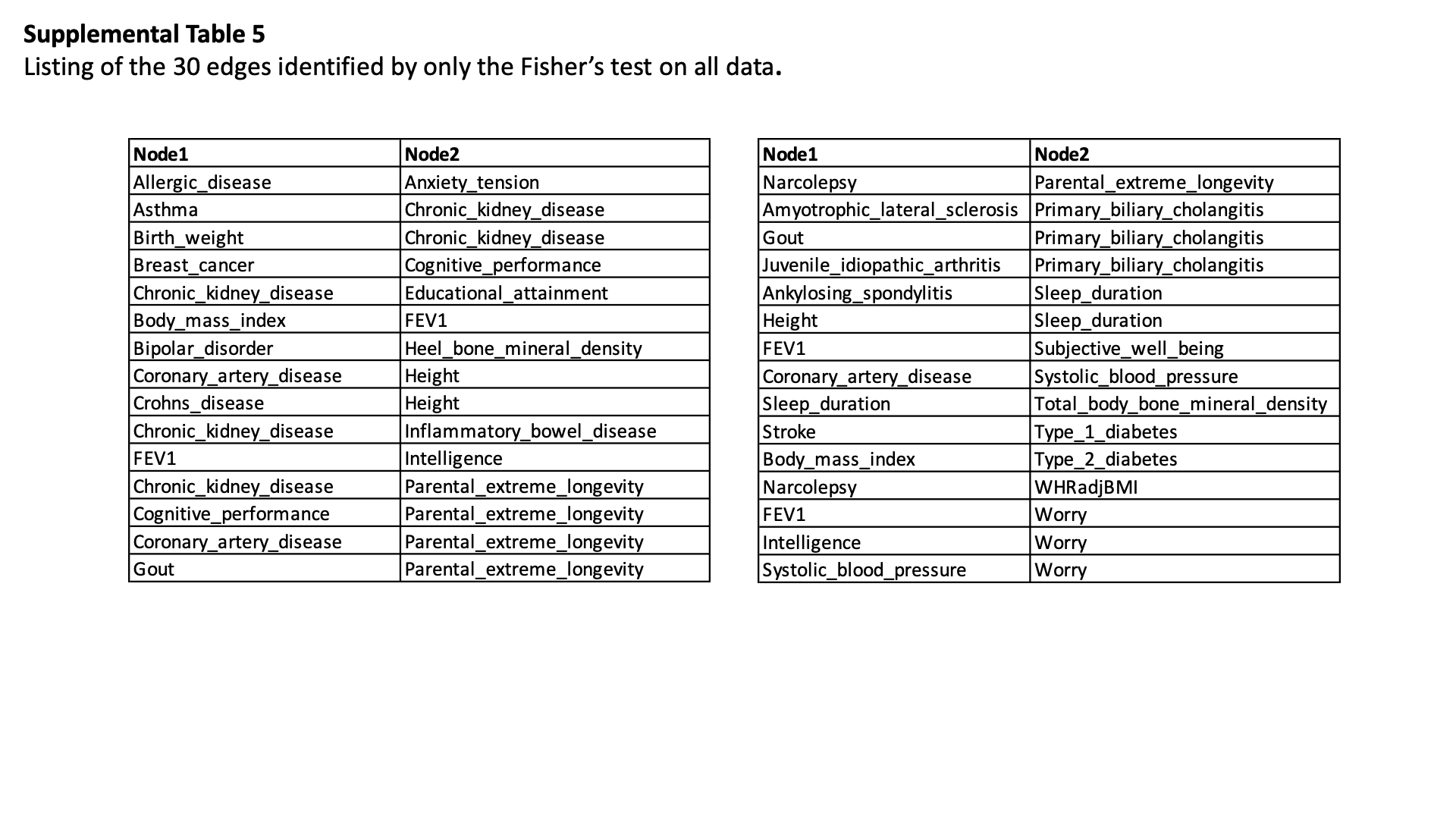

Supplement: Supplementary file 3 [file Supplementaryfile5.tiff]

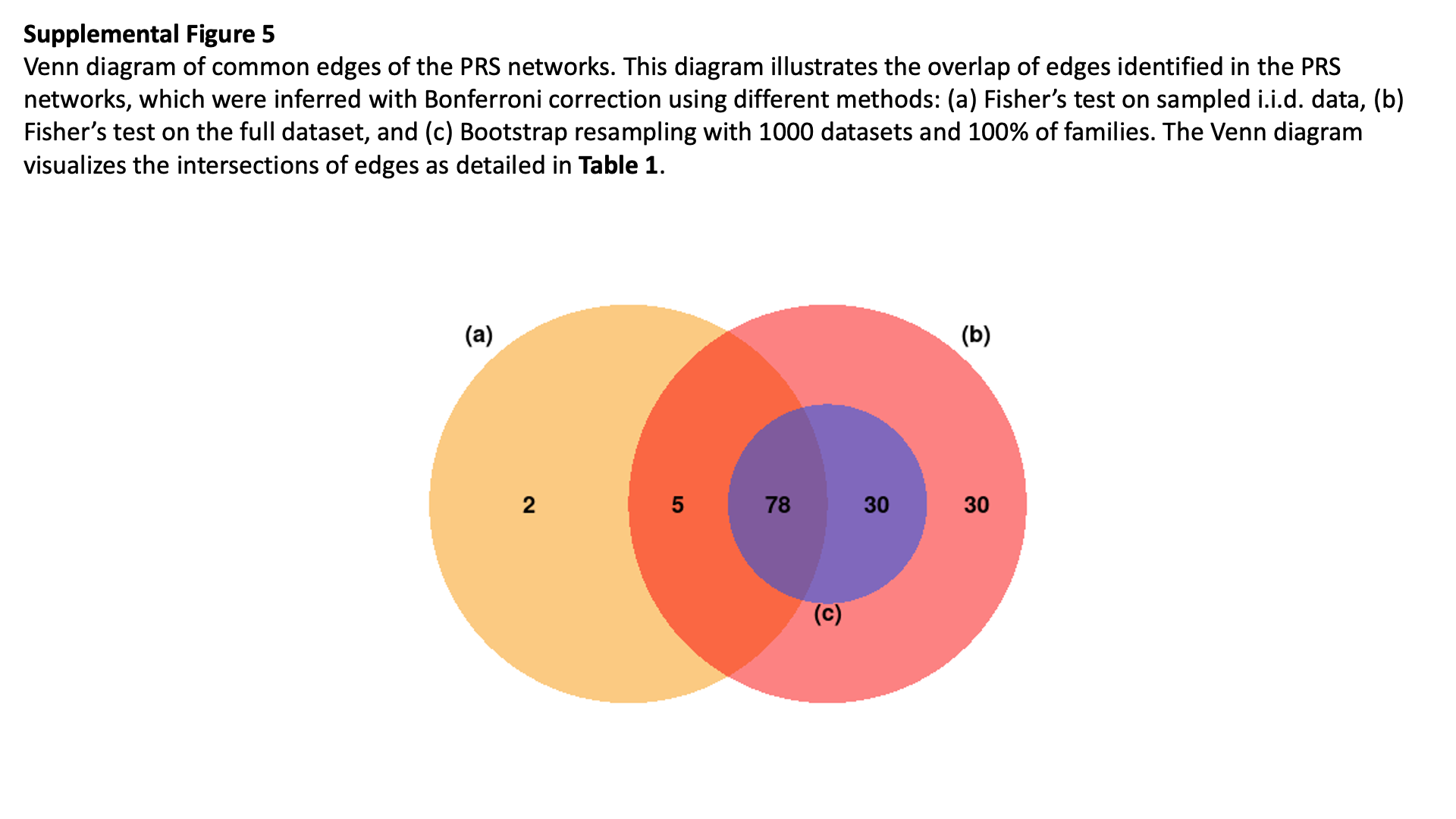

Supplement: Supplementary file 4 [file Image5.tiff]

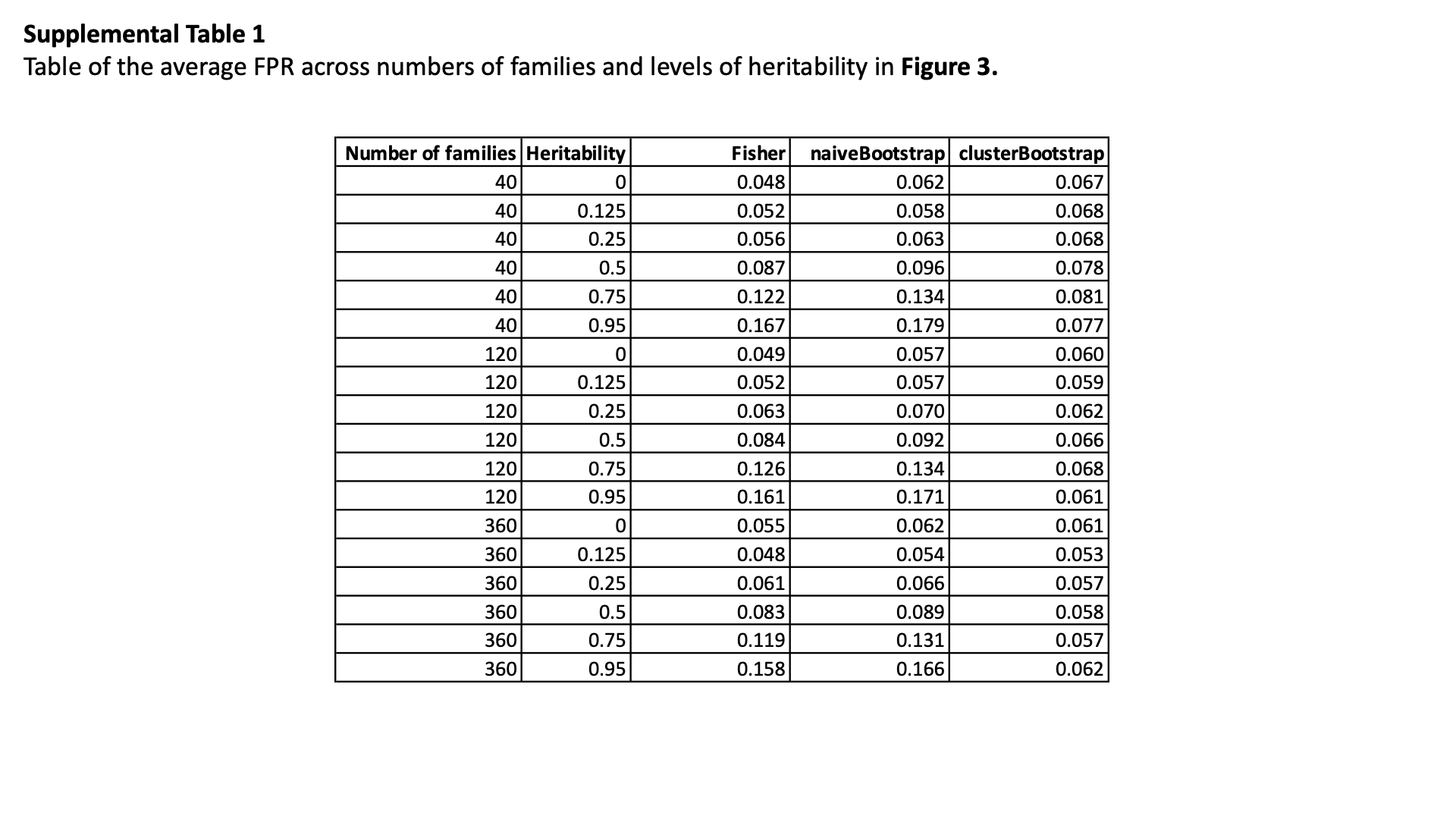

Supplement: Supplementary file 5 [file Supplementaryfile1.tiff]

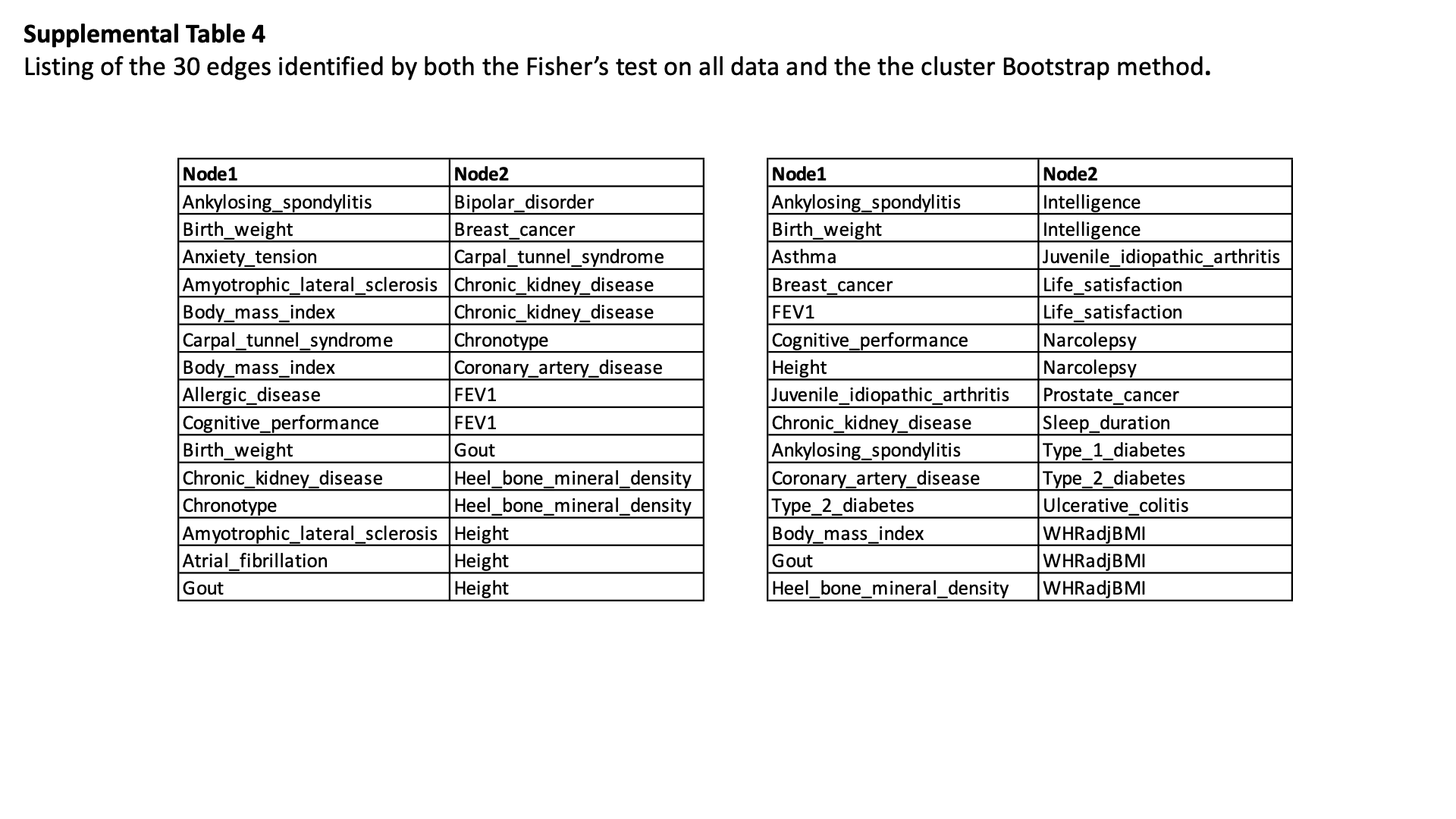

Supplement: Supplementary file 6 [file Supplementaryfile4.tiff]

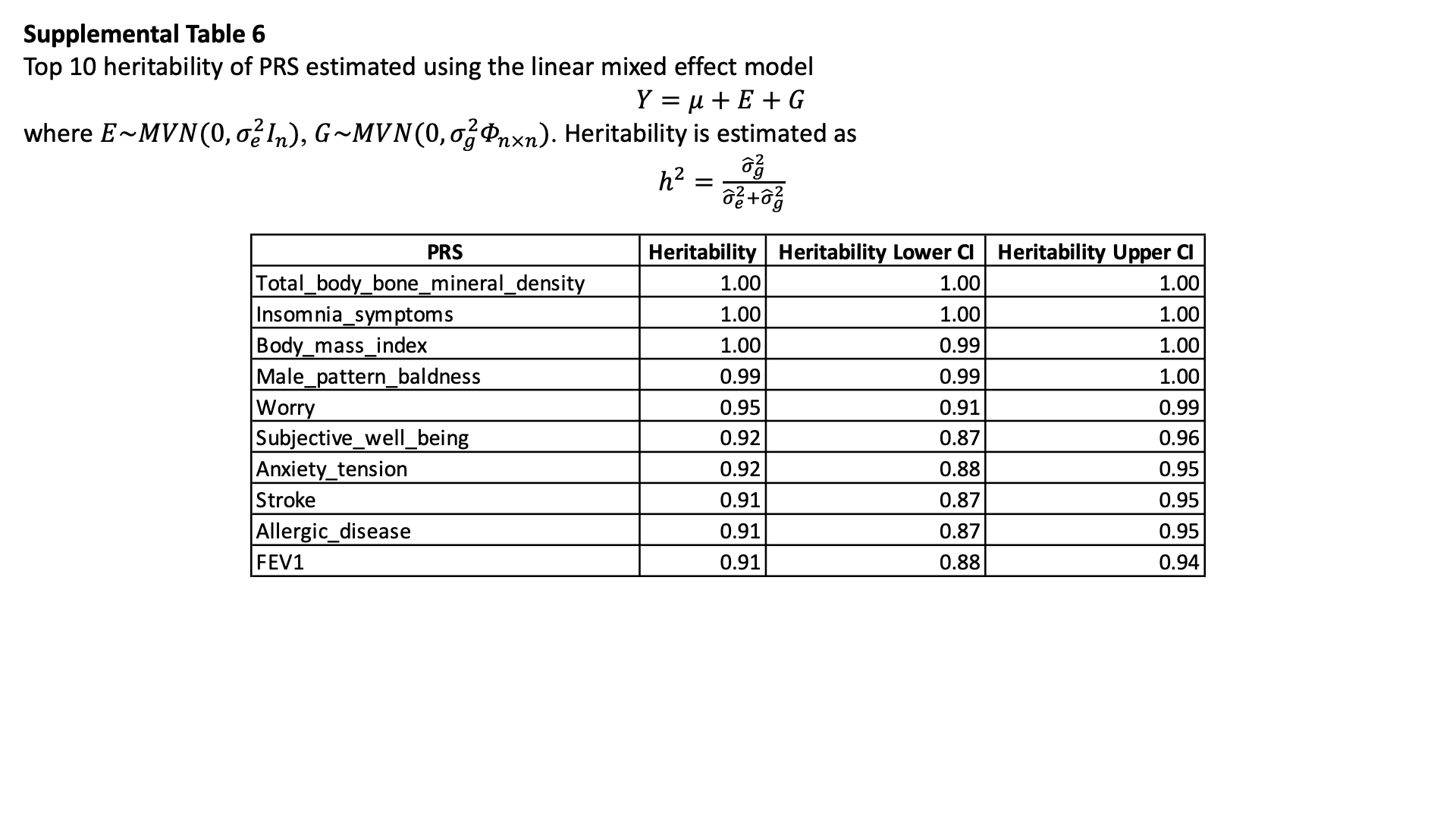

Supplement: Supplementary file 7 [file Supplementaryfile6.tiff]

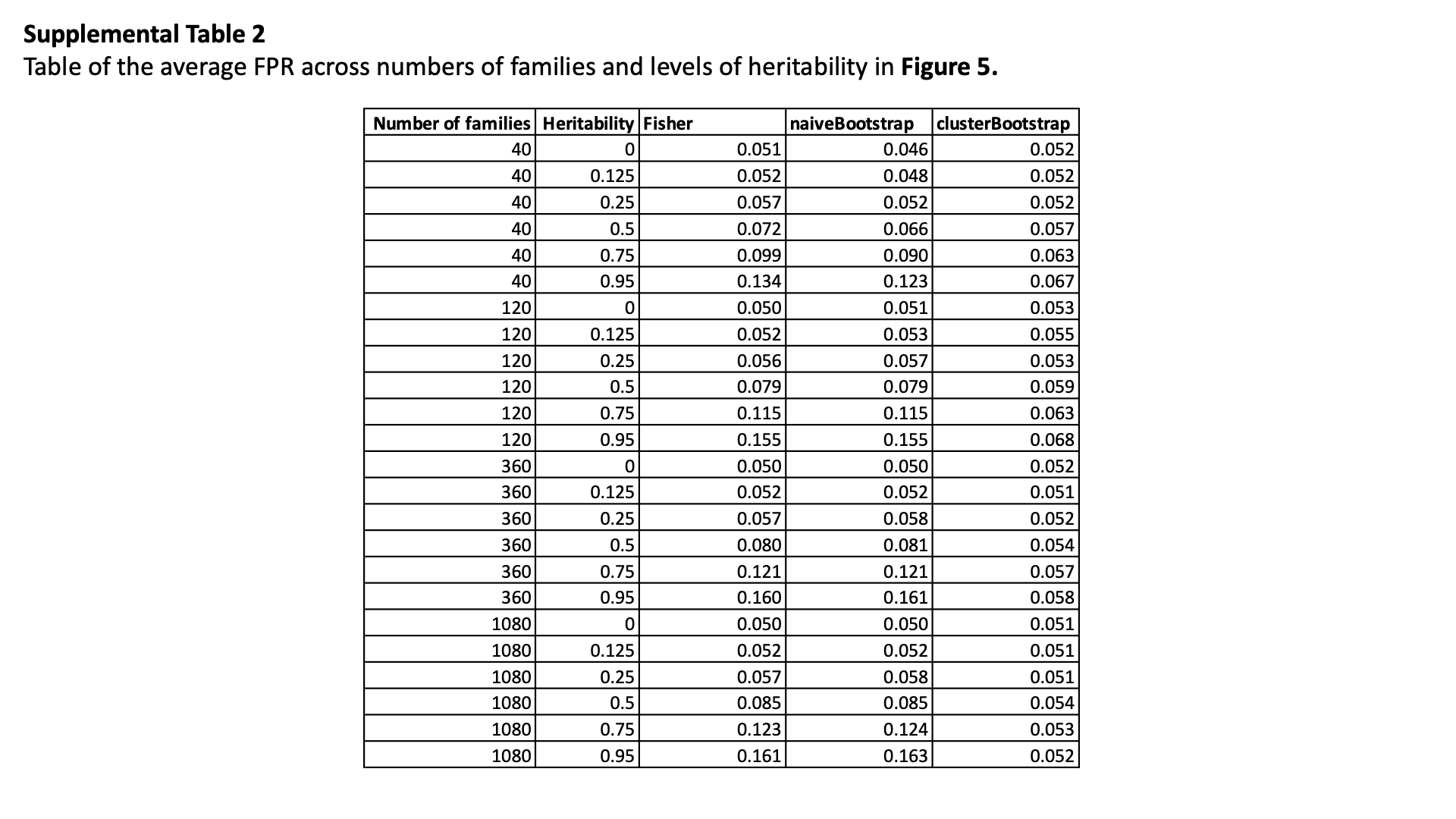

Supplement: Supplementary file 8 [file Supplementaryfile2.tiff]

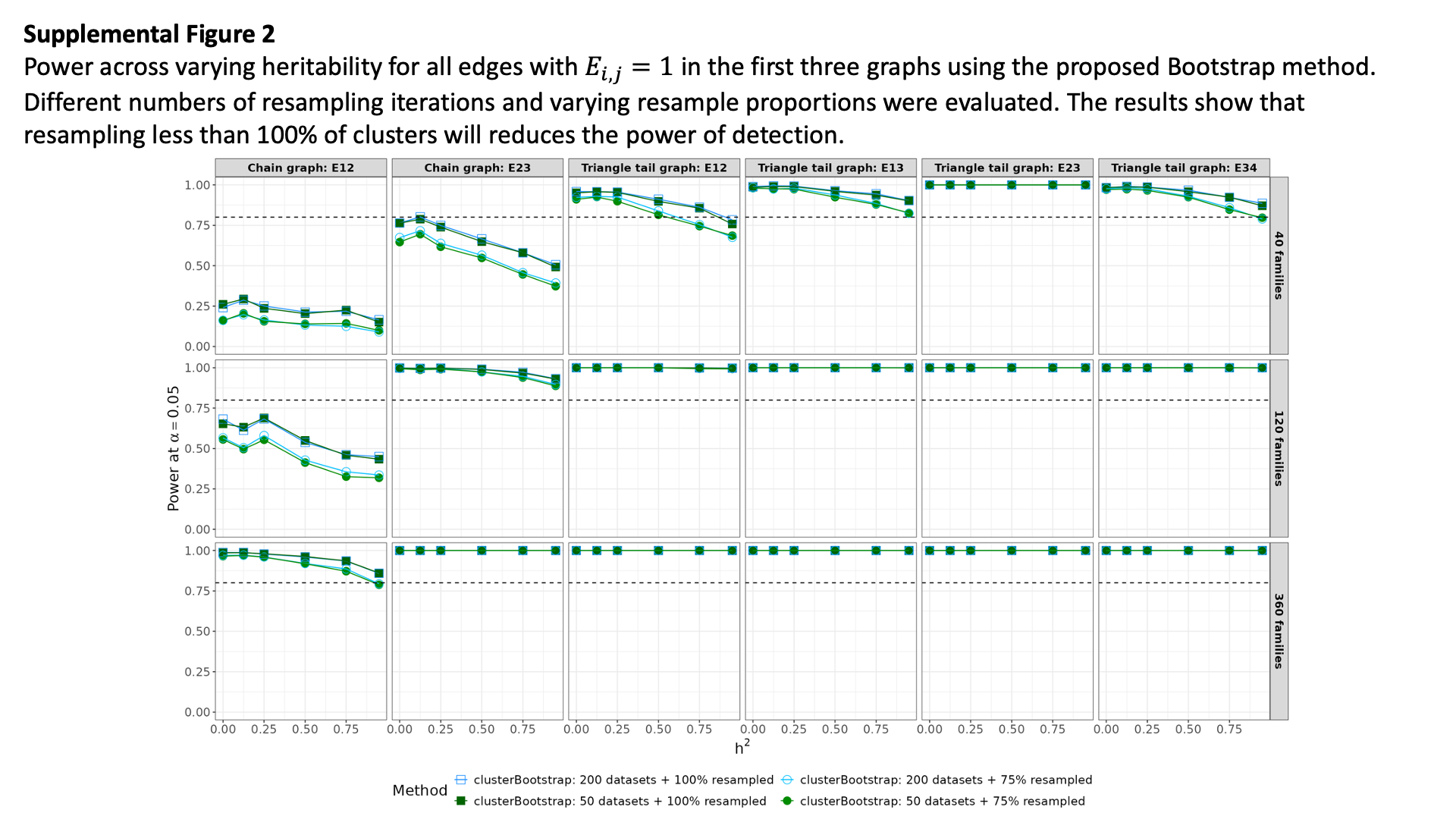

Supplement: Supplementary file 9 [file Image2.tiff]

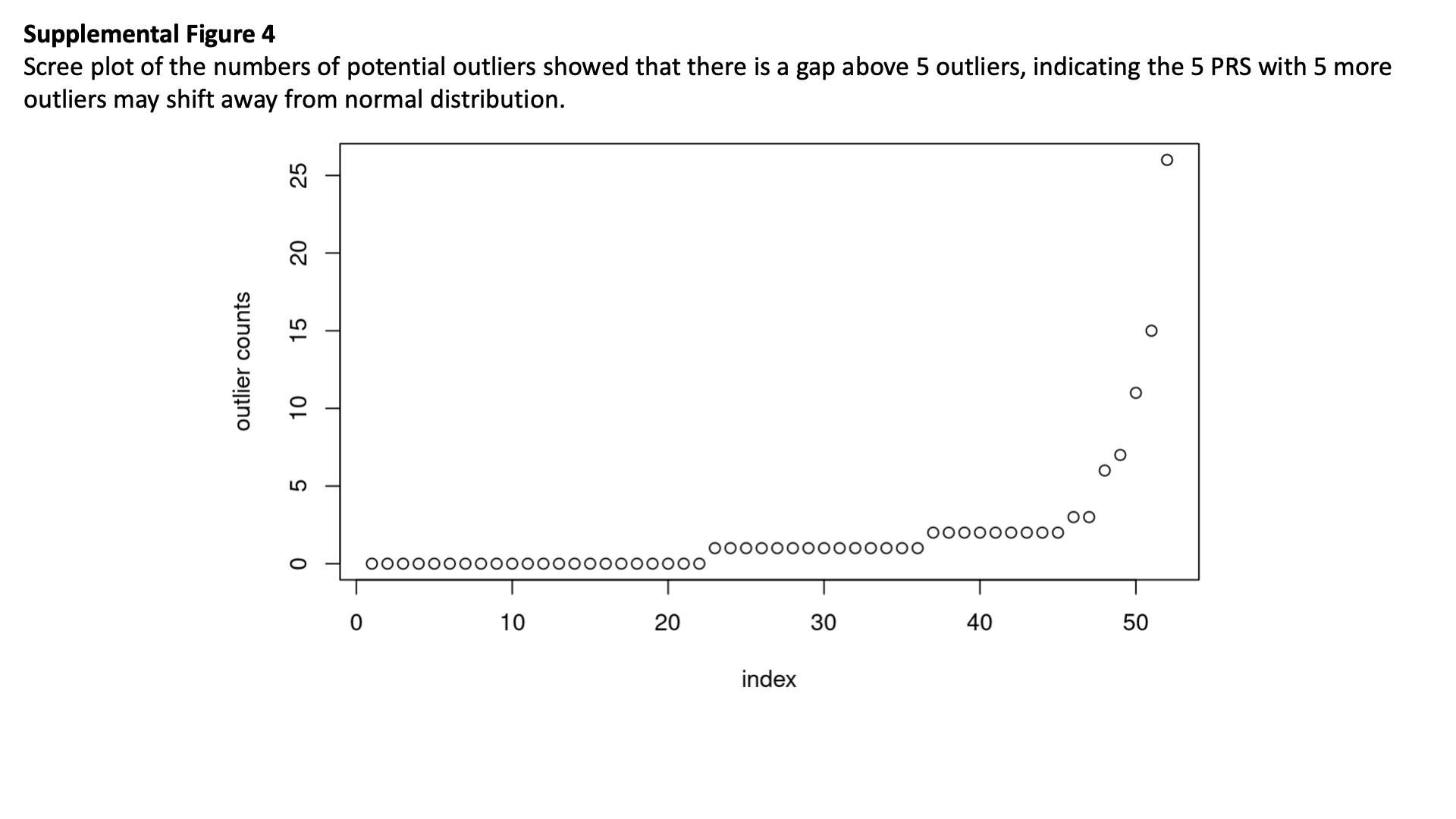

Supplement: Supplementary file 10 [file Image4.tiff]

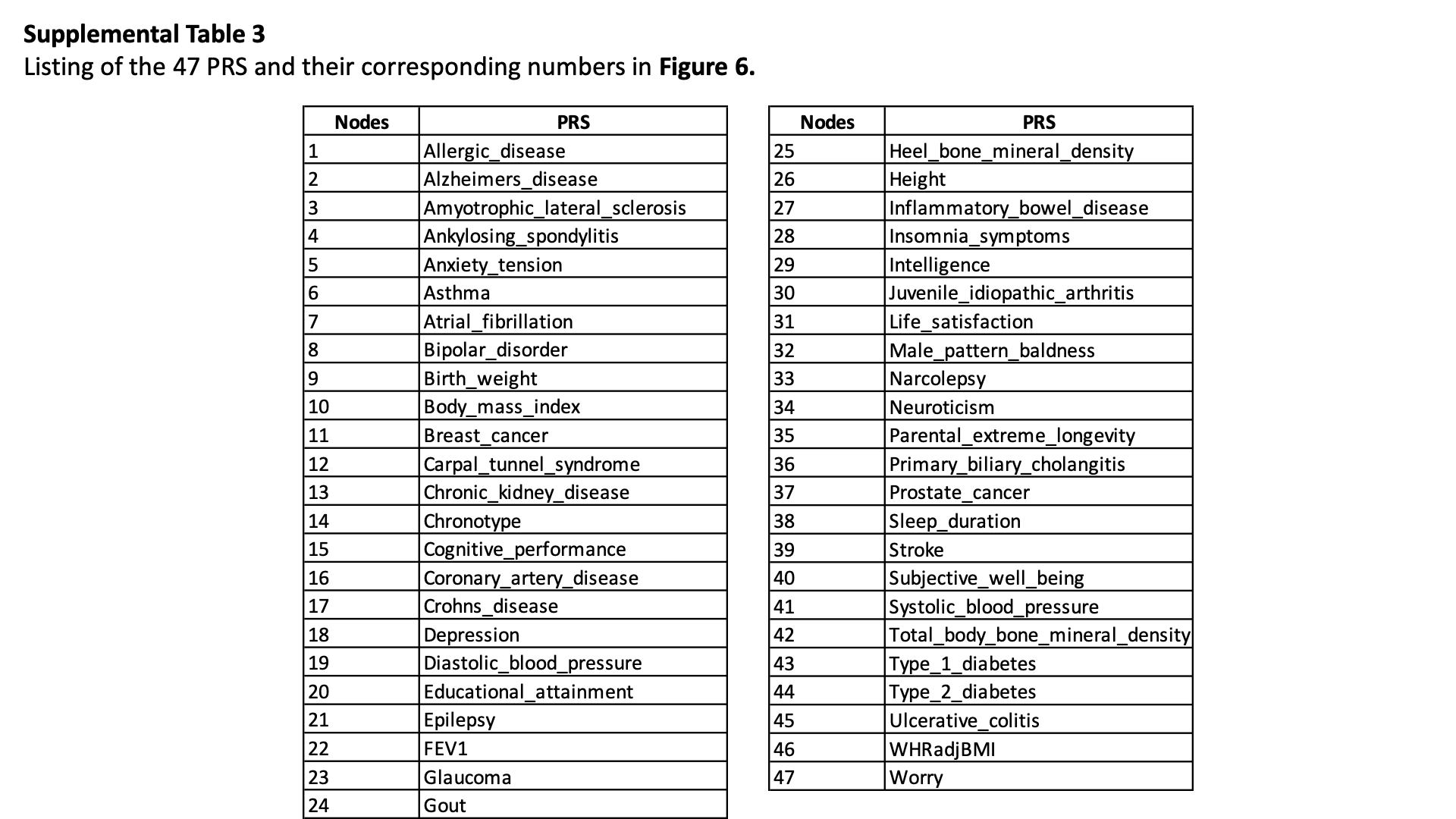

Supplement: Supplementary file 11 [file Supplementaryfile3.tiff]
